# The mitochondrial genome sequence of tachinid fly, *Hemyda hertingi,* from China

**DOI:** 10.1128/mra.00460-25

**Published:** 2025-09-08

**Authors:** Peng Hou, Li Liu, Xin Ma, Jianfeng Wang, Qiang Wang

**Affiliations:** 1 College of Life Science and Bioengineering, Shenyang University 47824, Shenyang, China; 2 Shanghai International Travel and Health Care Center 726364 , Shanghai, China; University of California Riverside, Riverside, California, USA

**Keywords:** Tachinidae, *Hemyda hertingi*, mitochondrial genome, China

## Abstract

Tachinid flies act as key biological vectors in elucidating plant–insect–microbe dynamic interactions. We report the mitochondrial genome sequence of *Hemyda hertingi* from China. The mitogenome spans 14,775 base pairs in length, with a GC content of 21.2%, and contains 13 protein-coding genes, 22 tRNAs, 2 rRNAs, and 1 non-coding region.

## ANNOUNCEMENT

Tachinid fly is the most rapidly evolving and species-rich groups within the Diptera (1). The larvae are predominantly parasitic, while adults are primarily phytophagous (2). As a crucial vector for microbial transmission, the study of this insect holds significant value for biological pest control and symbiont research (3). Clarifying its genetic information and sequence structure is pivotal for exploring plant–insect–microbe interaction mechanisms (4). However, molecular genetic analyses of *Hemyda hertingi* remain unexplored. This study reports the mitochondrial genome sequence of *H. hertingi*, providing genome sequence data for future research.

The specimens were collected from the Qianshan Mountains in Liaoning Province, China (41.0357N, 123.1378E), morphology identified by Wang et al. (5), and deposited in 85% ethanol at −20℃ for subsequent DNA isolation. Following the manufacturer’s protocol, whole genome DNA isolation was performed on the muscle tissue of an adult leg using the DNeasy DNA Extraction Kit (TIANGEN, Beijing, China). The integrity of extracted DNA was evaluated by 1% agarose gel electrophoresis. Purity and concentration were quantified using a UV-3200PCS spectrophotometer (MAPADA, Shanghai, China). Presence of mitochondrial DNA was confirmed through polymerase chain reaction amplification with universal COI gene primers (mLCOIinF: 5′-GWACWGGWTGAACWGTWTAYCCYCC-3′, dgHCO2198: 5′-TAAACYTCAGGRTGACCRAAYCA-3′). NGS library preparation was performed using the Illumina TruSeq DNA Sample Prep Kit (Illumina, San Diego, CA, USA). Sequencing was conducted on the Illumina NovaSeq 6000 platform (Berry Genomic, Beijing, China), generating 150 bp paired-end reads in length. This yielded 44,079,270 raw reads, totaling 10.5 Gb of sequencing data with an average coverage of 50×. The raw data quality control and filtering were performed using fastp v0.36 (6) and included adapter trimming, sliding-window quality trimming (Q < 20), and removal of reads containing ambiguous bases, yielding clean reads. *De novo* mitochondrial genome assembly was conducted using MITObim v1.9.1 (7), employing the mitogenome of a closely related species (*Ectophasia rotundiventris*, GenBank accession number MK644821) as a bait reference. Following three iterative cycles, yielding a 14,775 bp contig mitochondrial genome.

Gene annotation was performed using Geneious Prime v10.3 (8) with the mitochondrial genetic code tTable 5(transl_table = 5). Gene boundaries were manually corrected and optimized through comparative analysis using NCBI BLAST against the reference mitochondrial genomes of *Drino* sp. and *Janthinomyia* sp. (GenBank accession numbers MK644820 and MK644822). The assembled genome exhibited sequence identity percentages of 96.3% and 96.7% with the identified circular mitochondrial genomes, respectively.

**Fig 1:**
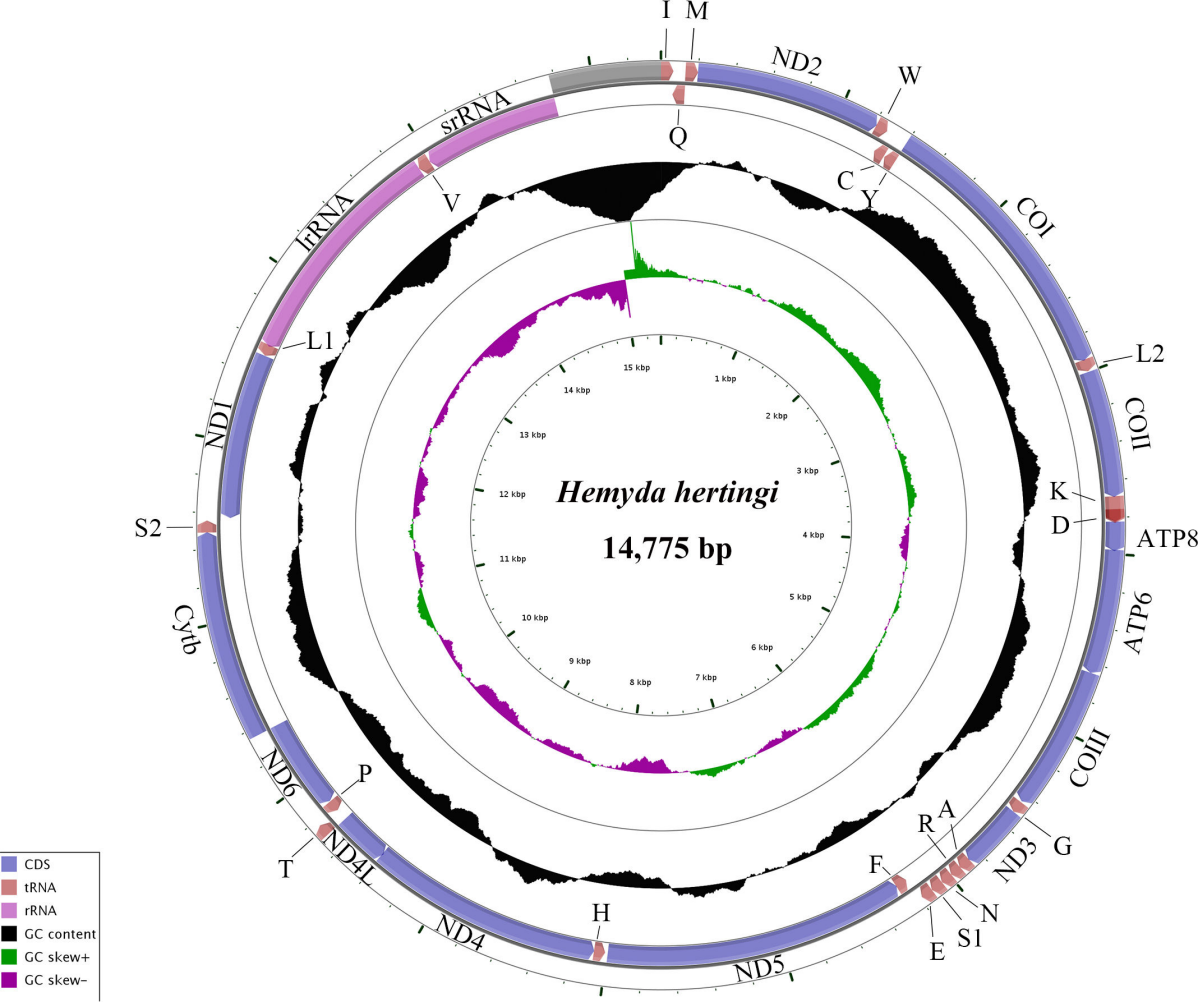
The mitogenome map of *H. hertingi*. The names of PCGs and rRNAs are indicated by standard abbreviations, while names of tRNAs are represented by a single-letter abbreviation. The first circle shows the gene arrangement, and arrows indicate the orientation of gene transcription. The second circle indicates the GC content, the third circle shows the GC skew, and the innermost circle shows the sequence length.

The mitogenome sequence of *H. hertingi* comprises a non-coding region, 2 rRNA genes, 13 protein-coding genes, and 22 tRNA genes, distributed as 23 genes on the heavy strand and 14 genes on the light strand (Table 1). The overall base composition was 40.8% A, 38.0% T, 12.7% C, and 8.5% G. The ATP6, ATP8, COI-III, CYTB, ND2, ND3, and ND6 genes are encoded by the heavy strand, while the light strand encodes ND1, ND4, ND4L, and ND5. Visualization using CG View server v1.0 (9) generated a circular genome map (Fig. 1), showing transcription directions, gene locations, and GC content.

**TABLE 1:** Mitochondrial genome content, organization, and codon information of *H. hertingi*

Gene	Location	Gene length (bp)	Start codon	Stop codon	Anti-codon	H/L strand^*b*^	Intergenic region length (bp)
trnI	1–67	67			GAT	+	0
trnQ	65–133	69			TTG	−	−3
trnM	133–200	68			CAT	+	−1
ND2	200–1210	1,011	ATT	TAA		+	−1
trnW	1210–1275	66			TCA	+	−1
trnC	1268–1329	62			GCA	+	−8
trnY	1335–1399	65			GTA	+	5
COI	1398–2936	1,539	TCG	TAA		+	−2
trnL	2932–2997	66			TAA	+	−5
COII	3002–3691	689	ATG	TAA		+	4
trnK	3699–3758	60			TTT	+	7
trnD	3758–3824	67			GTC	+	−1
ATP8	3825–3983	159	ATC	TAA		+	0
ATP6	3977–4654	678	ATG	TAA		+	−7
COIII	4654–5442	789	ATG	TAA		+	−1
trnG	5449–5513	65			TCC	+	6
ND3	5514–5867	354	ATT	TAG		+	0
trnA	5866–5930	65			TGC	+	−2
trnR	5930–5991	62			TCG	+	−1
trnN	5992–6056	65			GTT	+	0
trnS	6057–6124	68			GCT	+	0
trnE	6125–6190	66			TTC	+	0
trnF	6209–6274	66			GAA	−	18
ND5	6273–7991	1,719	ATA	T^*a*^		−	−2
trnH	8007–8070	64			GTG	−	15
ND4	8070–9407	1,338	TAT	T^*a*^		−	−1
ND4L	9403–9699	297	TTA	T^*a*^		−	−5
trnT	9702–9766	65			TGT	+	2
trnP	9766–9830	65			TGG	−	−1
ND6	9834–10358	525	ATT	TAA		+	3
CYTB	10358–11494	1,137	ATG	TAG		+	−1
trnS	11493–11557	65			TGA	+	−2
ND1	11573–12520	948	TTG	TAA		−	15
trnL	12522–12586	65			TAG	−	1
rrnL	12578–13916	1,339				−	−9
trnV	13917–13988	72			TAC	−	0
rrnS	13989–14775	787				−	0

^*a*^ Truncated termination codon.

^*b*^ Heavy (H) strand = “+”, Light (L) strand = “−”.

This study provides the mitochondrial genome sequence of *H. hertingi* from China, contributing valuable data for genetic and evolutionary studies of this vector insect and resource species.

## Data Availability

The complete mitochondrial genome sequence of Hemyda hertingi has been deposited in the DDBJ/ENA/GenBank database under the accession number PV250220. The version described in this manuscript is the first version, PV250220.1. The raw sequencing data from this study have been deposited in the NCBI Sequence Read Archive (SRA) under the accession number SRR34900917 (BioProject accession number PRJNA1246752 and BioSample accession number SAMN47793021).
